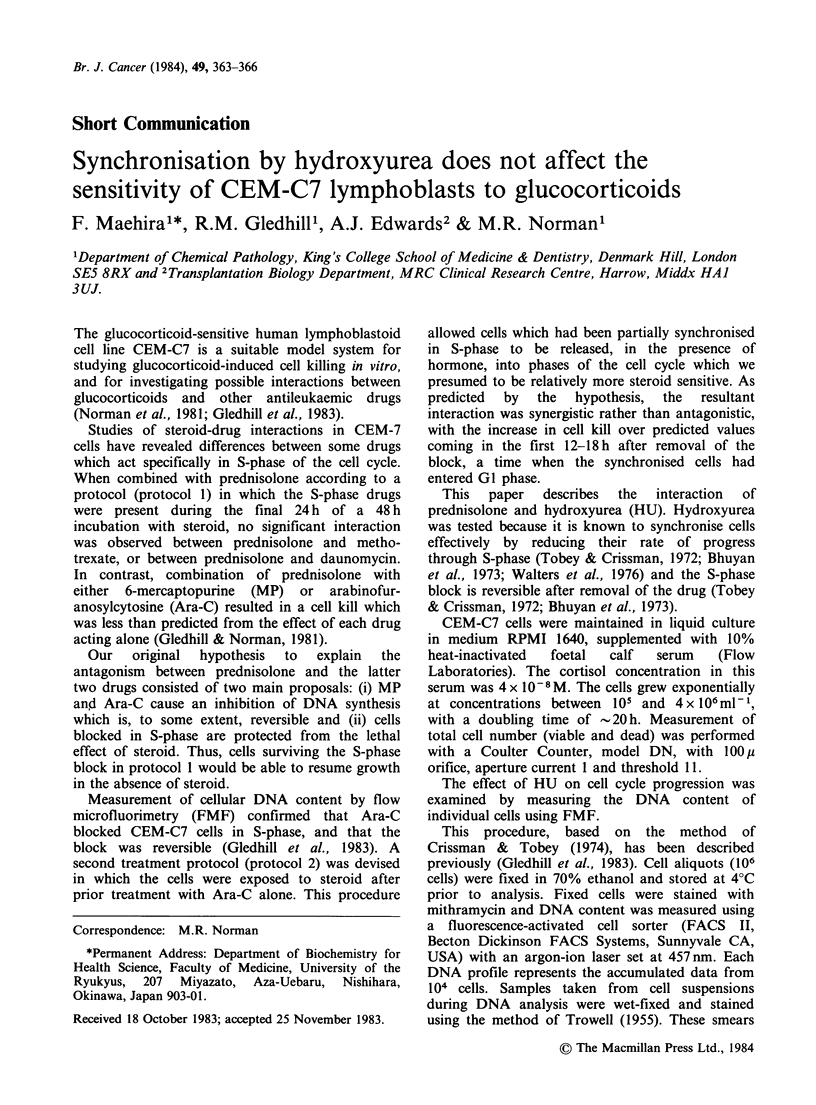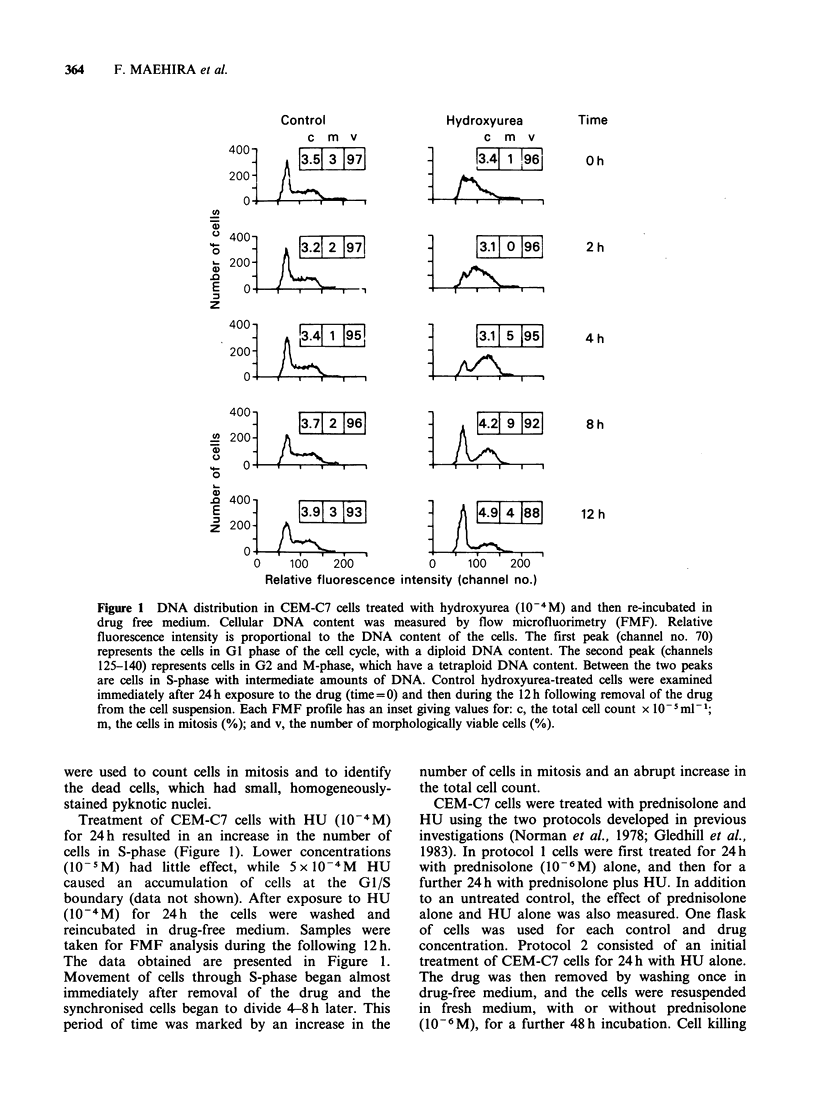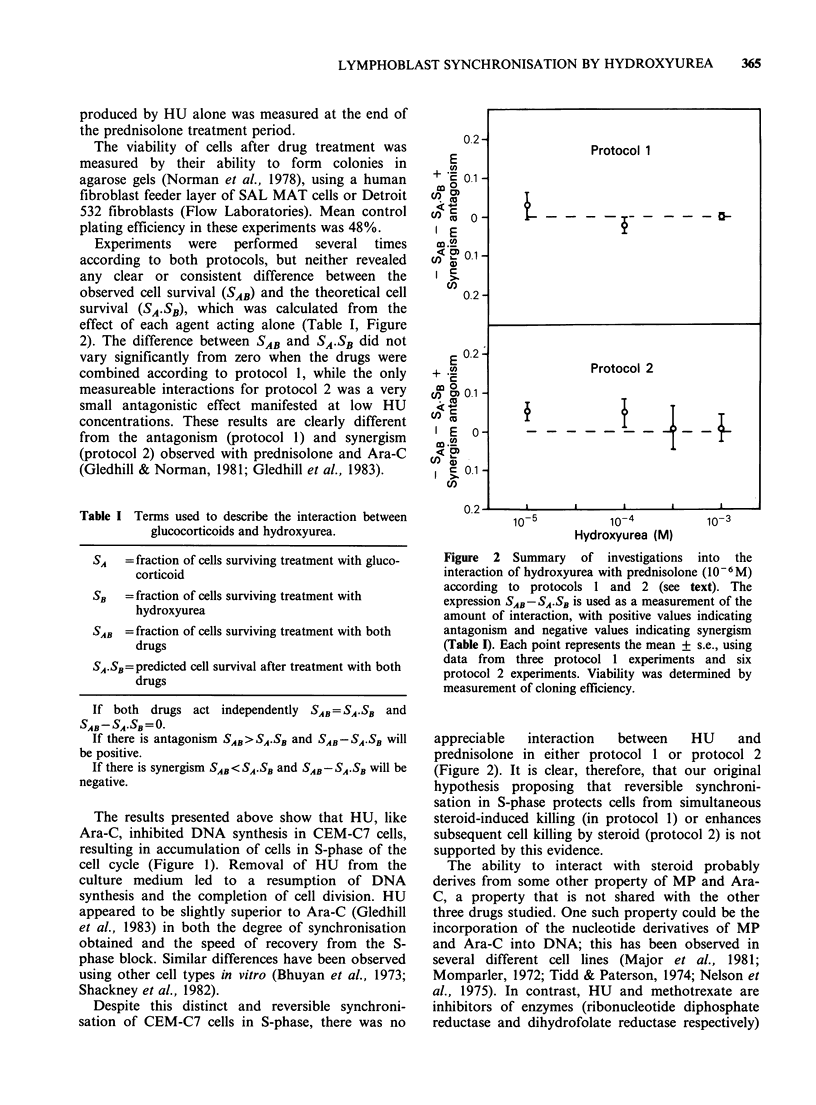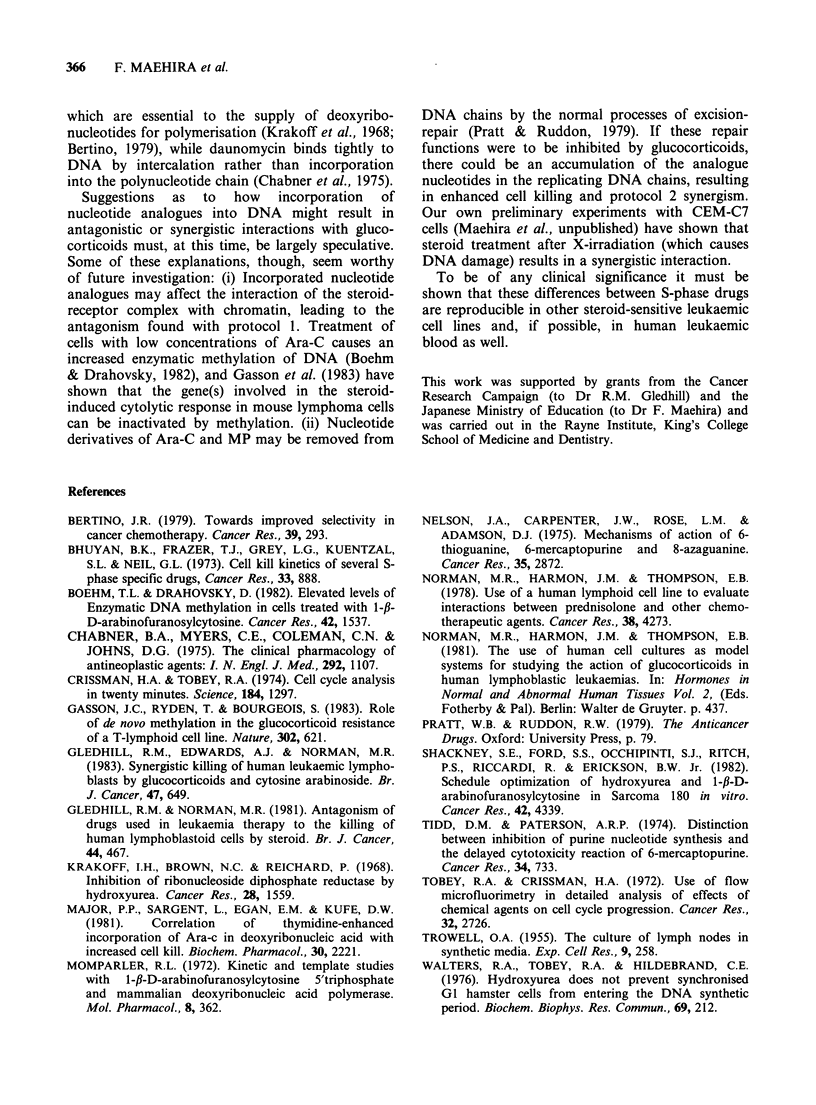# Synchronisation by hydroxyurea does not affect the sensitivity of CEM-C7 lymphoblasts to glucocorticoids.

**DOI:** 10.1038/bjc.1984.57

**Published:** 1984-03

**Authors:** F. Maehira, R. M. Gledhill, A. J. Edwards, M. R. Norman


*Br. J. Cancer* (1984), **49**, 363–366

### Short Communication

### Synchronisation by hydroxyurea does not affect the sensitivity of CEM-C7 lymphoblasts to glucocorticoids

F. Maehira[1]*, R.M. Gledhill[1], A.J. Edwards[2] & M.R. Norman[1]

[1]*Department of Chemical Pathology, King's College School of Medicine & Dentistry, Denmark Hill, London SE5 8RX and* [2]*Transplantation Biology Department, MRC Clinical Research Centre, Harrow, Middx HA1 3UJ.*

The glucocorticoid-sensitive human lymphoblastoid cell line CEM-C7 is a suitable model system for studying glucocorticoid-induced cell killing *in vitro*, and for investigating possible interactions between glucocorticoids and other antileukaemic drugs (Norman *et al.*, 1981; Gledhill *et al.*, 1983).

Studies of steroid-drug interactions in CEM-7 cells have revealed differences between some drugs which act specifically in S-phase of the cell cycle. When combined with prednisolone according to a protocol (protocol 1) in which the S-phase drugs were present during the final 24 h of a 48 h incubation with steroid, no significant interaction was observed between prednisolone and methotrexate, or between prednisolone and daunomycin. In contrast, combination of prednisolone with either 6-mercaptopurine (MP) or arabinofuranosylcytosine (Ara-C) resulted in a cell kill which was less than predicted from the effect of each drug acting alone (Gledhill & Norman, 1981).

Our original hypothesis to explain the antagonism between prednisolone and the latter two drugs consisted of two main proposals: (i) MP and Ara-C cause an inhibition of DNA synthesis which is, to some extent, reversible and (ii) cells blocked in S-phase are protected from the lethal effect of steroid. Thus, cells surviving the S-phase block in protocol 1 would be able to resume growth in the absence of steroid.

Measurement of cellular DNA content by flow microfluorimetry (FMF) confirmed that Ara-C blocked CEM-C7 cells in S-phase, and that the block was reversible (Gledhill *et al.*, 1983). A second treatment protocol (protocol 2) was devised in which the cells were exposed to steroid after prior treatment with Ara-C alone. This procedure allowed cells which had been partially synchronised in S-phase to be released, in the presence of hormone, into phases of the cell cycle which we presumed to be relatively more steroid sensitive. As predicted by the hypothesis, the resultant interaction was synergistic rather than antagonistic, with the increase in cell kill over predicted values coming in the first 12–18 h after removal of the block, a time when the synchronised cells had entered G1 phase.

This paper describes the interaction of prednisolone and hydroxyurea (HU). Hydroxyurea was tested because it is known to synchronise cells effectively by reducing their rate of progress through S-phase (Tobey & Crissman, 1972; Bhuyan *et al.*, 1973; Walters *et al.*, 1976) and the S-phase block is reversible after removal of the drug (Tobey & Crissman, 1972; Bhuyan *et al.*, 1973).

CEM-C7 cells were maintained in liquid culture in medium RPMI 1640, supplemented with 10% heat-inactivated foetal calf serum (Flow Laboratories). The cortisol concentration in this serum was $4 \times 10^{-8}$ M. The cells grew exponentially at concentrations between $10^5$ and $4 \times 10^6 ml^{-1}$, with a doubling time of ~20 h. Measurement of total cell number (viable and dead) was performed with a Coulter Counter, model DN, with 100 $\mu$ orifice, aperture current 1 and threshold 11.

The effect of HU on cell cycle progression was examined by measuring the DNA content of individual cells using FMF.

This procedure, based on the method of Crissman & Tobey (1974), has been described previously (Gledhill *et al.*, 1983). Cell aliquots ($10^6$ cells) were fixed in 70% ethanol and stored at 4°C prior to analysis. Fixed cells were stained with mithramycin and DNA content was measured using a fluorescence-activated cell sorter (FACS II, Becton Dickinson FACS Systems, Sunnyvale CA, USA) with an argon-ion laser set at 457 nm. Each DNA profile represents the accumulated data from $10^4$ cells. Samples taken from cell suspensions during DNA analysis were wet-fixed and stained using the method of Trowell (1955). These smears

Correspondence: M.R. Norman

*Permanent Address: Department of Biochemistry for Health Science, Faculty of Medicine, University of the Ryukyus, 207 Miyazato, Aza-Uebaru, Nishihara, Okinawa, Japan 903-01.

Received 18 October 1983; accepted 25 November 1983.

© The Macmillan Press Ltd., 1984

**Figure 1** DNA distribution in CEM-C7 cells treated with hydroxyurea ($10^{-4}$ M) and then re-incubated in drug free medium. Cellular DNA content was measured by flow microfluorimetry (FMF). Relative fluorescence intensity is proportional to the DNA content of the cells. The first peak (channel no. 70) represents the cells in G1 phase of the cell cycle, with a diploid DNA content. The second peak (channels 125–140) represents cells in G2 and M-phase, which have a tetraploid DNA content. Between the two peaks are cells in S-phase with intermediate amounts of DNA. Control hydroxyurea-treated cells were examined immediately after 24 h exposure to the drug (time = 0) and then during the 12 h following removal of the drug from the cell suspension. Each FMF profile has an inset giving values for: c, the total cell count $\times 10^{-5}$ $ml^{-1}$; m, the cells in mitosis (%); and v, the number of morphologically viable cells (%).

were used to count cells in mitosis and to identify the dead cells, which had small, homogeneously-stained pyknotic nuclei.

Treatment of CEM-C7 cells with HU ($10^{-4}$ M) for 24 h resulted in an increase in the number of cells in S-phase (Figure 1). Lower concentrations ($10^{-5}$ M) had little effect, while $5 \times 10^{-4}$ M HU caused an accumulation of cells at the G1/S boundary (data not shown). After exposure to HU ($10^{-4}$ M) for 24 h the cells were washed and reincubated in drug-free medium. Samples were taken for FMF analysis during the following 12 h. The data obtained are presented in Figure 1. Movement of cells through S-phase began almost immediately after removal of the drug and the synchronised cells began to divide 4–8 h later. This period of time was marked by an increase in the number of cells in mitosis and an abrupt increase in the total cell count.

CEM-C7 cells were treated with prednisolone and HU using the two protocols developed in previous investigations (Norman *et al.*, 1978; Gledhill *et al.*, 1983). In protocol 1 cells were first treated for 24 h with prednisolone ($10^{-6}$ M) alone, and then for a further 24 h with prednisolone plus HU. In addition to an untreated control, the effect of prednisolone alone and HU alone was also measured. One flask of cells was used for each control and drug concentration. Protocol 2 consisted of an initial treatment of CEM-C7 cells for 24 h with HU alone. The drug was then removed by washing once in drug-free medium, and the cells were resuspended in fresh medium, with or without prednisolone ($10^{-6}$ M), for a further 48 h incubation. Cell killing produced by HU alone was measured at the end of the prednisolone treatment period.

The viability of cells after drug treatment was measured by their ability to form colonies in agarose gels (Norman *et al.*, 1978), using a human fibroblast feeder layer of SAL MAT cells or Detroit 532 fibroblasts (Flow Laboratories). Mean control plating efficiency in these experiments was 48%.

Experiments were performed several times according to both protocols, but neither revealed any clear or consistent difference between the observed cell survival ($S_{AB}$) and the theoretical cell survival ($S_A.S_B$), which was calculated from the effect of each agent acting alone (Table I, Figure 2). The difference between $S_{AB}$ and $S_A.S_B$ did not vary significantly from zero when the drugs were combined according to protocol 1, while the only measureable interactions for protocol 2 was a very small antagonistic effect manifested at low HU concentrations. These results are clearly different from the antagonism (protocol 1) and synergism (protocol 2) observed with prednisolone and Ara-C (Gledhill & Norman, 1981; Gledhill *et al.*, 1983).

**Table I** Terms used to describe the interaction between glucocorticoids and hydroxyurea.

| | |
|---|---|
| $S_A$ | =fraction of cells surviving treatment with glucocorticoid |
| $S_B$ | =fraction of cells surviving treatment with hydroxyurea |
| $S_{AB}$ | =fraction of cells surviving treatment with both drugs |
| $S_A.S_B$ | =predicted cell survival after treatment with both drugs |

If both drugs act independently $S_{AB}=S_A.S_B$ and $S_{AB}-S_A.S_B=0$.

If there is antagonism $S_{AB}>S_A.S_B$ and $S_{AB}-S_A.S_B$ will be positive.

If there is synergism $S_{AB}<S_A.S_B$ and $S_{AB}-S_A.S_B$ will be negative.

**Figure 2** Summary of investigations into the interaction of hydroxyurea with prednisolone ($10^{-6}$M) according to protocols 1 and 2 (see text). The expression $S_{AB}-S_A.S_B$ is used as a measurement of the amount of interaction, with positive values indicating antagonism and negative values indicating synergism (Table I). Each point represents the mean ± s.e., using data from three protocol 1 experiments and six protocol 2 experiments. Viability was determined by measurement of cloning efficiency.

The results presented above show that HU, like Ara-C, inhibited DNA synthesis in CEM-C7 cells, resulting in accumulation of cells in S-phase of the cell cycle (Figure 1). Removal of HU from the culture medium led to a resumption of DNA synthesis and the completion of cell division. HU appeared to be slightly superior to Ara-C (Gledhill *et al.*, 1983) in both the degree of synchronisation obtained and the speed of recovery from the S-phase block. Similar differences have been observed using other cell types *in vitro* (Bhuyan *et al.*, 1973; Shackney *et al.*, 1982).

Despite this distinct and reversible synchronisation of CEM-C7 cells in S-phase, there was no appreciable interaction between HU and prednisolone in either protocol 1 or protocol 2 (Figure 2). It is clear, therefore, that our original hypothesis proposing that reversible synchronisation in S-phase protects cells from simultaneous steroid-induced killing (in protocol 1) or enhances subsequent cell killing by steroid (protocol 2) is not supported by this evidence.

The ability to interact with steroid probably derives from some other property of MP and Ara-C, a property that is not shared with the other three drugs studied. One such property could be the incorporation of the nucleotide derivatives of MP and Ara-C into DNA; this has been observed in several different cell lines (Major *et al.*, 1981; Momparler, 1972; Tidd & Paterson, 1974; Nelson *et al.*, 1975). In contrast, HU and methotrexate are inhibitors of enzymes (ribonucleotide diphosphate reductase and dihydrofolate reductase respectively)

which are essential to the supply of deoxyribonucleotides for polymerisation (Krakoff *et al.*, 1968; Bertino, 1979), while daunomycin binds tightly to DNA by intercalation rather than incorporation into the polynucleotide chain (Chabner *et al.*, 1975).

Suggestions as to how incorporation of nucleotide analogues into DNA might result in antagonistic or synergistic interactions with glucocorticoids must, at this time, be largely speculative. Some of these explanations, though, seem worthy of future investigation: (i) Incorporated nucleotide analogues may affect the interaction of the steroid-receptor complex with chromatin, leading to the antagonism found with protocol 1. Treatment of cells with low concentrations of Ara-C causes an increased enzymatic methylation of DNA (Boehm & Drahovsky, 1982), and Gasson *et al.* (1983) have shown that the gene(s) involved in the steroid-induced cytolytic response in mouse lymphoma cells can be inactivated by methylation. (ii) Nucleotide derivatives of Ara-C and MP may be removed from DNA chains by the normal processes of excision-repair (Pratt & Ruddon, 1979). If these repair functions were to be inhibited by glucocorticoids, there could be an accumulation of the analogue nucleotides in the replicating DNA chains, resulting in enhanced cell killing and protocol 2 synergism. Our own preliminary experiments with CEM-C7 cells (Maehira *et al.*, unpublished) have shown that steroid treatment after X-irradiation (which causes DNA damage) results in a synergistic interaction.

To be of any clinical significance it must be shown that these differences between S-phase drugs are reproducible in other steroid-sensitive leukaemic cell lines and, if possible, in human leukaemic blood as well.

This work was supported by grants from the Cancer Research Campaign (to Dr R.M. Gledhill) and the Japanese Ministry of Education (to Dr F. Maehira) and was carried out in the Rayne Institute, King's College School of Medicine and Dentistry.

### References

BERTINO, J.R. (1979). Towards improved selectivity in cancer chemotherapy. *Cancer Res.*, **39**, 293.

BHUYAN, B.K., FRAZER, T.J., GREY, L.G., KUENTZAL, S.L. & NEIL, G.L. (1973). Cell kill kinetics of several S-phase specific drugs, *Cancer Res.*, **33**, 888.

BOEHM, T.L. & DRAHOVSKY, D. (1982). Elevated levels of Enzymatic DNA methylation in cells treated with 1-β-D-arabinofuranosylcytosine. *Cancer Res.*, **42**, 1537.

CHABNER, B.A., MYERS, C.E., COLEMAN, C.N. & JOHNS, D.G. (1975). The clinical pharmacology of antineoplastic agents: *I. N. Engl. J. Med.*, **292**, 1107.

CRISSMAN, H.A. & TOBEY, R.A. (1974). Cell cycle analysis in twenty minutes. *Science*, **184**, 1297.

GASSON, J.C., RYDEN, T. & BOURGEOIS, S. (1983). Role of *de novo* methylation in the glucocorticoid resistance of a T-lymphoid cell line. *Nature*, **302**, 621.

GLEDHILL, R.M., EDWARDS, A.J. & NORMAN, M.R. (1983). Synergistic killing of human leukaemic lymphoblasts by glucocorticoids and cytosine arabinoside. *Br. J. Cancer*, **47**, 649.

GLEDHILL, R.M. & NORMAN, M.R. (1981). Antagonism of drugs used in leukaemia therapy to the killing of human lymphoblastoid cells by steroid. *Br. J. Cancer*, **44**, 467.

KRAKOFF, I.H., BROWN, N.C. & REICHARD, P. (1968). Inhibition of ribonucleoside diphosphate reductase by hydroxyurea. *Cancer Res.*, **28**, 1559.

MAJOR, P.P., SARGENT, L., EGAN, E.M. & KUFE, D.W. (1981). Correlation of thymidine-enhanced incorporation of Ara-c in deoxyribonucleic acid with increased cell kill. *Biochem. Pharmacol.*, **30**, 2221.

MOMPARLER, R.L. (1972). Kinetic and template studies with 1-β-D-arabinofuranosylcytosine 5′triphosphate and mammalian deoxyribonucleic acid polymerase. *Mol. Pharmacol.*, **8**, 362.

NELSON, J.A., CARPENTER, J.W., ROSE, L.M. & ADAMSON, D.J. (1975). Mechanisms of action of 6-thioguanine, 6-mercaptopurine and 8-azaguanine. *Cancer Res.*, **35**, 2872.

NORMAN, M.R., HARMON, J.M. & THOMPSON, E.B. (1978). Use of a human lymphoid cell line to evaluate interactions between prednisolone and other chemotherapeutic agents. *Cancer Res.*, **38**, 4273.

NORMAN, M.R., HARMON, J.M. & THOMPSON, E.B. (1981). The use of human cell cultures as model systems for studying the action of glucocorticoids in human lymphoblastic leukaemias. In: *Hormones in Normal and Abnormal Human Tissues Vol. 2*, (Eds. Fotherby & Pal). Berlin: Walter de Gruyter. p. 437.

PRATT, W.B. & RUDDON, R.W. (1979). *The Anticancer Drugs*. Oxford: University Press, p. 79.

SHACKNEY, S.E., FORD, S.S., OCCHIPINTI, S.J., RITCH, P.S., RICCARDI, R. & ERICKSON, B.W. Jr. (1982). Schedule optimization of hydroxyurea and 1-β-D-arabinofuranosylcytosine in Sarcoma 180 *in vitro*. *Cancer Res.*, **42**, 4339.

TIDD, D.M. & PATERSON, A.R.P. (1974). Distinction between inhibition of purine nucleotide synthesis and the delayed cytotoxicity reaction of 6-mercaptopurine. *Cancer Res.*, **34**, 733.

TOBEY, R.A. & CRISSMAN, H.A. (1972). Use of flow microfluorimetry in detailed analysis of effects of chemical agents on cell cycle progression. *Cancer Res.*, **32**, 2726.

TROWELL, O.A. (1955). The culture of lymph nodes in synthetic media. *Exp. Cell Res.*, **9**, 258.

WALTERS, R.A., TOBEY, R.A. & HILDEBRAND, C.E. (1976). Hydroxyurea does not prevent synchronised G1 hamster cells from entering the DNA synthetic period. *Biochem. Biophys. Res. Commun.*, **69**, 212.